# Complicações Neurológicas em Pacientes com Endocardite Infecciosa: Perspectivas de um Centro Terciário

**DOI:** 10.36660/abc.20190586

**Published:** 2021-04-08

**Authors:** Sofia Alegria, Ana Marques, Inês Cruz, Ana Luísa Broa, Ana Rita F. Pereira, Isabel João, Otília Simões, Hélder Pereira

**Affiliations:** 1 Hospital Garcia de Orta EPE – Cardiologia Almada Portugal Hospital Garcia de Orta EPE – Cardiologia,^1^ Almada – Portugal.

**Keywords:** Endocardite Infeciosa/cirurgia, Endocardite Infecciosa/complicações, Sistema Nervoso Central/complicações, Acidente Vascular Cerebral, Embolização, Prognóstico, Mortalidade

## Abstract

**Fundamento:**

Complicações neurológicas são comuns em pacientes com endocardite infecciosa (EI). Dados recentes sugerem que os eventos neurológicos são os principais determinantes do prognóstico e que a cirurgia é crítica para melhorar o resultado.

**Objetivo:**

Caracterizar pacientes com EI e complicações neurológicas e determinar preditores de embolização para o sistema nervoso central (SNC) e mortalidade.

**Métodos:**

Análise retrospectiva de pacientes internados em centro terciário com diagnóstico de EI no período de 2006 a 2016. Significância estatística foi definida por um valor de p <0,05.

**Resultados:**

Identificamos 148 episódios de EI, 20% dos quais tinham evidências de embolização do SNC. Em pacientes com embolização do SNC, 76% apresentaram acidente vascular cerebral isquêmico. Durante o seguimento, 35% foram submetidos à cirurgia e a mortalidade hospitalar e em um ano foi de 39%. Esses pacientes tiveram hospitalizações mais longas, mas não houve diferenças significativas em relação à mortalidade em pacientes com e sem embolização do SNC. Os preditores independentes de complicações neurológicas foram diabetes (p = 0,005) e ausência de febre na apresentação (p = 0,049). A cirurgia foi associada a menor mortalidade (0 vs. 58%; p = 0,003), enquanto os pacientes com choque séptico tiveram pior prognóstico (75 vs. 25%; p = 0,014). Na regressão multivariada de Cox, a infecção pelo vírus da imunodeficiência humana (HIV) foi o único preditor independente de mortalidade hospitalar e de 1 ano (p = 0,011 em ambos).

**Conclusões:**

Nessa população, a embolização para o SNC foi comum, mais frequentemente apresentada como acidente vascular cerebral isquêmico, e esteve associada a maior tempo de internação, embora sem diferenças significativas na mortalidade. Nos pacientes com embolização do SNC, os submetidos à cirurgia tiveram boa evolução clínica, enquanto os pacientes com choque séptico e infecção pelo HIV tiveram pior evolução. Esses resultados devem ser interpretados com cautela, levando em consideração que os pacientes com complicações mais graves ou mais frágeis foram provavelmente menos considerados para a cirurgia, resultando em viés de seleção.

## Introdução

Complicações neurológicas são ocorrências comuns na endocardite infecciosa (EI), presente em 15-30% dos pacientes.^1^^–^^3^ A apresentação clínica é variável e pode incluir vários sintomas ou sinais, embora os sinais focais sejam predominantes e os acidentes vasculares isquêmicos diagnosticados com mais frequência. Também são observados: ataque isquêmico transitório, hemorragia intracerebral ou subaracnoide, abscesso cerebral, meningite e encefalopatia tóxica, e há fortes evidências de que embolias cerebrais clinicamente silenciosas adicionais ocorrem em 35-60% dos pacientes com EI.^4^^–^^6^ A encefalopatia relacionada à sepse, definida por confusão aguda ou delírio, com vários níveis de consciência, também pode contribuir para as manifestações neurológicas de EI.^7^

Diante disso, deve-se sempre considerar a EI no diagnóstico diferencial de um paciente que apresente evento cerebral agudo e sinais de infecção sistêmica ou história de síndrome febril indeterminada, lembrando que o diagnóstico precoce e a implementação de antibioticoterapia adequada podem reduzir o risco de embolização recorrente.^1^

Os fatores de risco para embolização do sistema nervoso central (SNC) são bem conhecidos e incluem tamanho e mobilidade da vegetação,^2,8-10^ infecção por *Staphylococcus aureus*^11^ e envolvimento da válvula mitral.^10^ No entanto, o risco de eventos embólicos do SNC na EI diminui drasticamente após o início de terapia antimicrobiana eficaz para menos de 1,71/1.000 pacientes-dia na segunda semana.^12^

As manifestações neurológicas ocorrem antes ou no momento do diagnóstico da EI na maioria dos casos, mas eventos novos ou recorrentes também podem ocorrer mais tarde no curso da EI. As complicações neurológicas estão associadas ao excesso de mortalidade, assim como às sequelas, principalmente no caso de acidente vascular cerebral^2^^,^^13^ e afetam tanto a terapia medicamentosa^14^ quanto o momento ideal para a cirurgia.^15^ O diagnóstico rápido e o início de terapias antibióticas apropriadas são de grande importância para prevenir uma complicação neurológica primária ou recorrente.^12^ A cirurgia precoce em pacientes de alto risco é o segundo pilar da prevenção de embolia, enquanto os medicamentos antitrombóticos não têm função.^1^ Dados recentes sugerem que os eventos neurológicos são um importante determinante do prognóstico e que a cirurgia tem um papel central na otimização do resultado.

No entanto, a ocorrência de complicações neurológicas levanta questões quanto ao momento da cirurgia, uma vez que a segurança da circulação extracorpórea em pacientes com esses eventos permanece controversa. A decisão deve ser individualizada após avaliação multidisciplinar, envolvendo cardiologistas, cirurgiões cardíacos, neurologistas e especialistas em doenças infecciosas. Se possível, a cirurgia deve ser adiada em pacientes com eventos isquêmicos grandes ou eventos hemorrágicos. Foi sugerido que a cirurgia deve ser considerada nas primeiras 72 horas em pacientes com eventos isquêmicos e insuficiência cardíaca grave, caso contrário, após quatro semanas. A cirurgia precoce parece segura em pacientes com ataques isquêmicos transitórios ou eventos silenciosos.

Portanto, o objetivo deste estudo foi caracterizar pacientes com EI e complicações neurológicas e determinar preditores de embolização para o SNC e de mortalidade associada.

## Métodos

Estudo retrospectivo e observacional baseado na análise dos prontuários de pacientes internados em um centro terciário de 500 leitos sem cirurgia cardíaca *in loco* e com diagnóstico de EI no período de 2006 a 2016. Foi realizada uma comparação entre pacientes com e sem complicações neurológicas. Este estudo foi aprovado pelo Comitê de Ética da instituição.

### Desenho do Estudo e Pacientes

Várias variáveis foram analisadas para o presente estudo, incluindo a data do diagnóstico da EI; idade e sexo do paciente; fatores de risco; tipo de endocardite (válvula nativa, válvula protética ou associada a dispositivo); válvulas afetadas; micro-organismos infecciosos; data, tipo e recorrência de complicações neurológicas; realização de cirurgia; e resultados. A endocardite protética da válvula foi considerada precoce se ocorresse dentro de 1 ano após o implante da válvula e tardia se ocorresse posteriormente.

Os episódios de EI foram avaliados retrospectivamente de acordo com os critérios de Duke modificados. Foram incluídos apenas pacientes com critérios para EI definida ou possível EI e nenhuma outra explicação para o quadro clínico. As recidivas foram consideradas como um único episódio, enquanto episódios distintos ocorrendo em um único paciente foram incluídos. A ecocardiografia transtorácica foi realizada em todos os pacientes, enquanto a ecocardiografia transesofágica foi realizada na maioria dos deles. As informações microbiológicas foram obtidas a partir de culturas de sangue e amostras de tecido cardíaco intraoperatórias, bem como de estudos sorológicos no caso de hemoculturas negativas.

### Definições

As complicações neurológicas foram classificadas nas seguintes categorias: complicações isquêmicas, hemorragia cerebral, aneurisma micótico, meningite e abscesso cerebral. O diagnóstico de complicações isquêmicas e hemorrágicas baseou-se em dados clínicos e radiológicos, derivados de tomografia computadorizada (TC) de crânio ou ressonância magnética (RM), realizados de acordo com a prática clínica. O diagnóstico de aneurisma micótico também foi apoiado por angiotomografia de crânio.

### Indicação para Cirurgia

A indicação de cirurgia cardíaca foi determinada pelos médicos assistentes de acordo com as diretrizes da *European Society of Cardiology.*^1^ Todos os pacientes com indicação de cirurgia eram discutidos pela equipe cardíaca (incluindo cardiologistas, cirurgiões cardíacos e, quando considerado necessário, outras especialidades, como neurologistas e especialistas em doenças infecciosas), sendo tomada a decisão sobre a realização e o momento da cirurgia. Quando indicada, a cirurgia foi realizada em centro de referência em cirurgia cardíaca definido pelo sistema nacional de saúde (Serviço de Cirurgia Cardíaca, Hospital Universitário de Santa Maria, CHULN, Lisboa, Portugal).

## Análise Estatística

As variáveis contínuas são descritas como média ± desvio padrão ou mediana e intervalo interquartil (IQR), de acordo com a avaliação da normalidade com o teste de Kolmogorov-Smirnov. Variáveis categóricas são relatadas como porcentagens e números absolutos. A comparação entre as variáveis em diferentes grupos de pacientes foi realizada com o teste X^2^ de Pearson para variáveis categóricas ou o teste *t* de amostras independentes ou o teste U de Mann-Whitney para variáveis contínuas. A análise das características basais, tipo de endocardite, etiologia, complicações e tratamento foi realizada por episódio, enquanto a análise da mortalidade foi realizada por paciente.

Variáveis associadas ou com tendência de associação com embolização do SNC (p <0,10) foram testadas através de regressão logística uni- e multivariada, a fim de identificar preditores independentes de embolização na população geral. Na amostra de pacientes com embolização do SNC, as variáveis associadas ou com tendência a associação com mortalidade intra-hospitalar e em um ano (p <0,10) foram testadas com regressão de Cox univariada e multivariada por método *stepwise*, para identificar preditores independentes de prognóstico. As curvas de sobrevida de Kaplan Meier foram usadas para identificar preditores de desfecho, que foram comparados com o teste de log-rank.

Todos os testes foram bilaterais e a significância estatística foi definida como p <0,05. A análise estatística foi realizada no IBM SPSS Statistics, versão 24.0 (IBM Corporation, Armonk, NY, EUA).

## Resultados

Identificamos 148 episódios de EI (ocorrendo em 142 pacientes; quatro pacientes tiveram dois episódios e um paciente teve três episódios; as recidivas foram consideradas como um único episódio). O total de episódios está detalhado na Tabela 1. O acompanhamento médio foi de 161 dias (IQR 34-1.358).

**Tabela 1:** Caracterização de todos os episódios de internações por endocardite (n = 148)

Característica	Episódios gerais (n=148)	Com embolização do SNC (n=29)	Sem embolização do SNC (n=119)	p*
Idade (anos) – mediana (IQR)	64 (51-75)	65 (53-69)	63 (50-75)	0,631
Idade ≤ 75 anos – n (%)	117 (79,1)	27 (93,1)	90 (75,6)	0,038
Sexo masculino – n (%)	111 (75,0)	22 (75,9)	89 (74,8)	0,905
**Histórico prévio – n (%)**				
Doença valvular cardíaca conhecida	72 (49,0)	15 (51,7)	57 (48,3)	0,741
Hipertensão arterial	76 (51,4)	16 (55,2)	60 (50,4)	0,646
Diabetes mellitus	28 (19,2)	11 (37,9)	17 (14,5)	0,004
Doença arterial coronariana	21 (14,2)	6 (20,7)	15 (12,6)	0,263
Insuficiência cardíaca	40 (27,0)	4 (13,8)	36 (30,3)	0,074
Doença renal crônica	22 (14,9)	4 (13,8)	18 (15,1)	0,856
Usuários de drogas intravenosas	19 (12,9)	3 (10,3)	16 (13,6)	0,644
Infecção por HIV	20 (13,6)	2 (6,9)	18 (15,3)	0,240
Procedimento invasivo nos 3 meses anteriores	54 (45,0)	10 (43,5)	44 (45,4)	0,870
**Tipo de endocardite - n (%)**				
Endocardite associada a cuidados de saúde	34 (23,3)	9 (31,0)	25 (21,4)	0,270
Endocardite de válvula protética	37 (25,0)	9 (31,0)	28 (23,5)	0,403
Endocardite de dispositivo cardíaco implantado	5 (3,4)	1 (3,4)	4 (3,4)	0,981
Válvula afetada – n (%)				
Aórtica	84 (56,8)	19 (65,5)	65 (54,6)	0,288
Mitral	58 (39,2)	13 (44,8)	45 (37,8)	0,488
Tricúspide	20 (13,5)	0 (0,0)	20 (16,8)	0,018
**Sintomas na apresentação – n (%)**				
Febre	102 (70,3)	16 (55,2)	86 (74,1)	0,045
Sopro cardíaco	81 (56,3)	14 (50,0)	67 (57,8)	0,458
**Micro-organismo – n (%)**				
Staphylococcus sp	49 (33,1)	8 (27,6)	41 (34,5)	0,481
Staphylococcus aureus	36 (24,3)	6 (20,7)	30 (25,2)	0,611
Streptococcus sp	43 (29,1)	9 (31,0)	34 (28,6)	0,793
Streptococcus bovis	14 (9,5)	3 (10,3)	11 (9,2)	0,856
Grupo Streptococcus viridans	18 (12,2)	2 (6,9)	16 (13,4)	0,333
Enterococcus sp	18 (12,2)	3 (10,3)	15 (12,6)	0,738
Bactérias Gram negativas	6 (4,1)	1 (3,4)	5 (4,2)	0,854
Fungi	3 (2,0)	1 (3,4)	2 (1,7)	0,545
EINH	30 (20,3)	6 (20,7)	24 (20,2)	0,950
**Complicações – n (%)**				
Abscesso perivalvular	20 (14,8)	4 (14,8)	16 (14,8)	1,000
Pseudoaneurisma	7 (5,2)	2 (7,4)	5 (4,6)	0,560
Fístula	6 (4,4)	1 (3,7)	5 (4,6)	0,835
Insuficiência cardíaca aguda	71 (48,0)	15 (51,7)	56 (47,1)	0,652
Choque séptico	31 (20,9)	8 (27,6)	23 (19,3)	0,327
**Tratamento**				
Cirurgia – n (%)	48 (32,4)	10 (34,5)	38 (31,9)	0,793
Duração da hospitalização (dias) – mediana (IQR)	40 (26-54)	51 (36-59)	38 (25-52)	0,011

* comparação entre pacientes com e sem embolização do SNC. SNC: sistema nervoso central; IQR: intervalo interquartil; HIV: vírus da imunodeficiência humana; EINH: endocardite infecciosa negativa para hemocultura.

Cerca de um terço deles (34,5%; n = 51) apresentava evidências de embolização sistêmica, sendo o local mais frequente o SNC (19,6%; n = 29). Outros locais de embolização incluíram a circulação periférica (4,1%, n = 6), os pulmões (2,7%, n = 4), as artérias coronárias (1,4%, n = 2) e o baço (1,4%, n = 2). No entanto, apenas 34,5% (n = 51) realizaram TC ou RM de crânio, de modo que a verdadeira incidência de embolização do SNC poderia ser subestimada. Considerando apenas os pacientes com EI do lado esquerdo, a incidência de embolização do SNC foi de 24,2% (n = 29). A caracterização dos pacientes com diagnóstico de embolização do SNC também está detalhada na Tabela 1. Esses pacientes eram predominantemente do sexo masculino, com mediana de idade de 65 anos; 48,3% tinham doença valvar previamente conhecida, 10,3% eram usuários de drogas intravenosas e 6,9% tinham infecção pelo vírus da imunodeficiência humana (HIV). A endocardite valvar nativa foi a mais comum (69,0%, n = 20), enquanto a endocardite valvar protética ocorreu em 31,0%, com 33,3% das próteses (n = 3) implantadas nos últimos 12 meses.

Entre os pacientes com infecção pelo HIV, 47,4% (n = 9) foram tratados com terapia antirretroviral, a contagem média de CD4 foi de 202,5 células/l (intervalo interquartil 10 - 402,5 células/µl), 62,5% (n = 10) tinham carga viral indetectável (carga viral mediana de 0 cópias/ml; intervalo interquartil 0 – 3.127 cópias/ml) e 46,7% (n = 7) atendiam critérios para a síndrome da imunodeficiência adquirida (AIDS).

**Figura 1:**
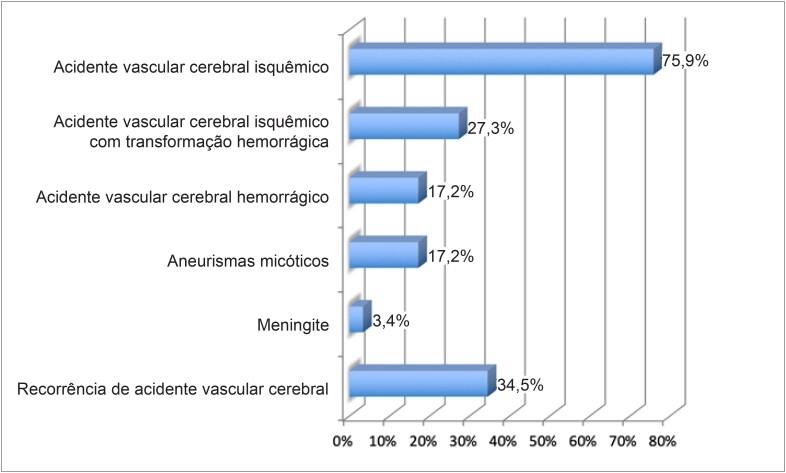
Complicações neurológicas em pacientes com endocardite (n = 29).

Os pacientes com embolização do SNC apresentaram acidente vascular cerebral isquêmico em 75,9% (n = 22) dos casos (com transformação hemorrágica em 27,3%; n = 6), acidente vascular cerebral hemorrágico em 17,2% (n = 5), aneurisma micótico em 17,2% (n = 5), e meningite em 3,4% (n = 1). Na admissão, os sintomas neurológicos estavam presentes em 41,4% (n = 12), e houve recorrência de acidente vascular cerebral (AVC – incluindo isquêmico e hemorrágico) em 34,5% (n = 10) (Figura 1).

### Preditores de Embolização do SNC

Pacientes com embolização do SNC, em comparação com aqueles sem, eram mais propensos a ter menos de 75 anos, ter diabetes e apresentar-se sem febre (Tabela 1). Além disso, nenhum paciente com embolização do SNC teve envolvimento das válvulas do lado direito, e suas hospitalizações foram mais longas (mediana de 51 *vs.* 38 dias). Não houve diferenças significativas em relação ao agente etiológico, ao envolvimento da válvula aórtica ou mitral, à proporção de pacientes submetidos à cirurgia ou ao desfecho.

Na regressão logística multivariada, os preditores independentes de embolização do SNC foram diabetes e ausência de febre (razão de risco – RR 3,78 e 2,41, respectivamente) (Tabela 2).

**Tabela 2:** Preditores de embolização do sistema nervoso central

Característica	RR	IC 95%	p
Diabetes mellitus	3,8	1,5-9,6	0,005
Ausência de febre na apresentação	2,4	1,0-5,8	0,049

RR: razão de risco; IC: intervalo de confiança.

### Resultados em Pacientes com Complicações Neurológicas

Durante o acompanhamento (mediana de 493 dias, IQR 36-1.863), 34,5% dos pacientes com embolização do SNC (n = 10) foram submetidos a cirurgias. O tempo médio da admissão à cirurgia foi de 41 dias (IQR 33-55) e do diagnóstico da complicação neurológica à cirurgia foi de 36 dias (IQR 28-43). A mortalidade hospitalar e a mortalidade em 1 ano foram de 39,3% (n = 11) e a mortalidade por todas as causas durante o seguimento foi de 46,4% (n = 13) (Tabela 3).

**Tabela 3:** Mortalidade de pacientes com endocardite (n=142)

Característica – n (%)	População geral (n=142)	Pacientes com embolização do SNC (n=29)	Pacientes sem embolização do SNC (n=113)	p*
Mortalidade hospitalar	43 (30,3)	11 (39,3)	32 (28,1)	0,247
Mortalidade de um ano	55 (38,7)	11 (39,3)	44 (38,6)	0,947
Mortalidade geral	64 (45,1)	13 (46,4)	51 (44,7)	0,872

* comparação entre pacientes com e sem embolização do SNC. SNC: sistema nervoso central.

**Figura 2:**
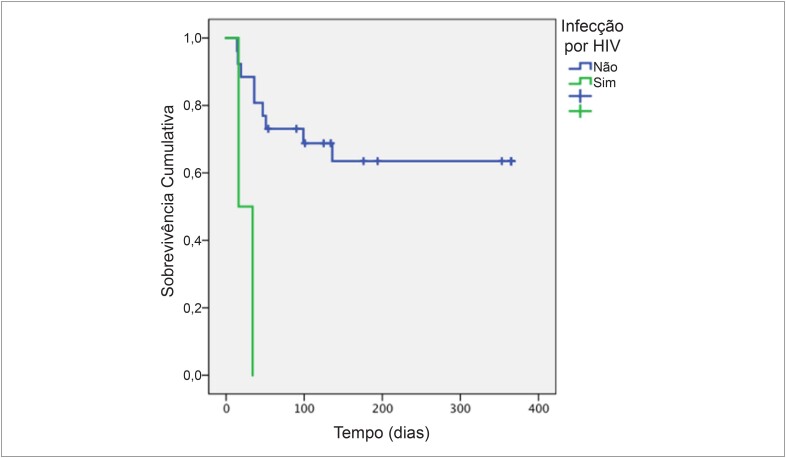
Curva de sobrevida de Kaplan-Meier de pacientes com endocardite e complicações neurológicas de acordo com o estado de infecção pelo vírus da imunodeficiência HIV: vírus da imunodeficiência humana.

A cirurgia foi associada à redução da mortalidade, tanto intra-hospitalar quanto em 1 ano (mortalidade em 1 ano em pacientes submetidos à cirurgia: 0 vs. 57,9%; p = 0,002). Além da cirurgia, as demais variáveis associadas à mortalidade intra-hospitalar foram a ocorrência de choque séptico e procedimentos invasivos nos últimos três meses (Tabela 4). Na análise de regressão multivariada de Cox, a infecção por HIV foi o único preditor independente de mortalidade hospitalar e de um ano (RR 10,5 e 10,6, respectivamente) (Tabelas 5 e 6, Figura 2).

**Tabela 4:** Associações com mortalidade hospitalar em pacientes com embolização do sistema nervoso central

Característica	OR	IC 95%	p
Diabetes mellitus	3,9	0,8-20,0	0,094
Infecção por HIV	N/A	N/A	0,068
Procedimento invasivo nos 3 meses anteriores	4,5	0,7-27,7	0,096
Choque séptico	9,0	1,4-59,8	0,014
Cirurgia	N/A	N/A	0,003

OR: odds ratio; IC: intervalo de confiança; HIV: vírus da imunodeficiência humana; N/A: não aplicável.

**Tabela 5:** Preditores independentes de mortalidade hospitalar em pacientes com embolização do sistema nervoso central

Característica	RR	IC 95%	p
Infecção por HIV	10,5	1,7-64,2	0,011

RR: razão de risco; IC: intervalo de confiança; HIV: vírus da imunodeficiência humana.

**Tabela 6:** Preditores independentes de mortalidade em um ano em pacientes com embolização do sistema nervoso central

Característica	RR	IC 95%	p
Infecção por HIV	10,6	1,7-64,8	0,011

RR: razão de risco; IC: intervalo de confiança; HIV: vírus da imunodeficiência humana.

## Discussão

Este estudo retrospectivo observacional descreve a incidência de complicações neurológicas em uma coorte de pacientes com EI de um único centro durante um período de 10 anos.

Complicações neurológicas são uma característica comum e frequentemente predominante da EI^3^^,^^13^^,^^16^^–^^18^ e o advento da TC e da RM permite uma avaliação clínica mais confiável desses eventos. No entanto, existem poucos dados disponíveis sobre o risco de AVC recorrente, a melhor abordagem em relação à terapia antitrombótica ou as consequências da cirurgia precoce.^2^

A frequência geral de complicações neurológicas na coorte do presente estudo foi em torno de 20%, mantendo os resultados de outras grandes coortes.^19^^,^^20^ No presente estudo, também foi observado que pacientes mais velhos apresentaram taxas mais baixas desses eventos, conforme relatado anteriormente,^19^^,^^21^ embora a causa dessa redução não seja totalmente compreendida. O uso de terapia antiplaquetária^22^^,^^23^ (frequentemente prescrita em pacientes idosos), um hipotético declínio da função hemostática e um menor tamanho das vegetações nessa população são alguns dos motivos propostos,^17^ mas também é possível que esses eventos sejam simplesmente subdiagnosticados nessa população devido a sinais e sintomas clínicos leves.

Os exames de imagem da cabeça não foram realizados de maneira rotineira em todos os pacientes, e a verdadeira incidência de complicações isquêmicas está provavelmente subestimada na presente coorte. Estudos usando RM^6^^,^^24^ mostraram que embolizações cerebrais agudas são significativamente mais prevalentes do que foi relatado anteriormente em estudos baseados em achados clínicos e tomografia computadorizada (30% dos eventos não detectados). Levando isso em consideração, é possível que alguns idosos menos sintomáticos do presente estudo tenham sido erroneamente classificados como sem complicações neurológicas. No entanto, outros relatos^20^^,^^25^ mostraram que pequenas complicações isquêmicas não têm impacto na evolução de pacientes com EI e, portanto, as conclusões essenciais não seriam alteradas.

No presente estudo, os preditores de embolização do SNC foram o histórico de diabetes mellitus e a ausência de febre na apresentação. Vários estudos demonstraram que o envolvimento da válvula mitral e o tamanho da vegetação são importantes preditores de AVC,^10^^,^^17^^,^^19^^,^^26^^–^^29^ enquanto outros não confirmaram essa observação.^29^^–^^32^ Na presente coorte, o envolvimento da válvula mitral não foi associado a complicações neurológicas. O tamanho da vegetação não foi avaliado, pois as medidas não estavam disponíveis para todos os pacientes e também porque as medidas existentes não eram padronizadas. Alguns autores enfatizam a importância do tamanho da vegetação apenas quando outros fatores estão presentes, como a localização da válvula mitral e o *Staphylococcus aureus* como o agente etiológico da EI.^30^^–^^33^ Nesta coorte, é possível hipotetizar que a influência da localização e do tamanho da vegetação no desenvolvimento de eventos embólicos foi provavelmente superada por fatores que levam a um atraso no diagnóstico e início da terapia antibiótica, como a ausência de febre na apresentação. Até onde sabemos, o diabetes mellitus não foi identificado anteriormente como fator de risco para embolização do SNC em pacientes com EI, embora esteja associado a um pior prognóstico desta.^1^ No entanto, o diabetes está associado a um risco aumentado de eventos cerebrovasculares e imunossupressão, de modo que podemos especular que essa condição possa facilitar o crescimento das vegetações e aumentar a gravidade e o impacto clínico da embolização, quando essa complicação ocorrer.

O momento da cirurgia nesses pacientes ainda é motivo de debate. A cirurgia imediata para prevenção de eventos embólicos com base em um tamanho de vegetação acima de 10 milímetros foi proposta em estudos ecocardiográficos iniciais,^34^ mas taxas mais altas de recidiva e deiscência de prótese após a cirurgia quando o tratamento antimicrobiano não foi concluído permanecem uma preocupação. Nesse sentido, dois estudos recentes demonstraram que a cirurgia precoce diminui efetivamente a embolia sistêmica sem aumentar a taxa de recidiva de EI ou problemas relacionados à válvula protética em comparação ao tratamento convencional.^35^^,^^36^

Da mesma forma, há preocupação com o risco de comprometimento neurológico pós-operatório quando a cirurgia valvar é realizada logo após um episódio isquêmico ou hemorrágico, e a literatura contém resultados contraditórios. Alguns autores descobriram que o risco de exacerbação é baixo quando a cirurgia é realizada dentro de 72 horas,^37^ enquanto outros relataram que o risco é maior no início da cirurgia e diminui gradualmente à medida que aumenta o atraso entre o evento neurológico e a operação.^38^ Considerando a falta de estudos controlados, as recomendações são baseadas nos resultados de relatórios publicados, e o conselho geralmente aceito é atrasar a cirurgia por pelo menos duas semanas no caso de AVC isquêmico grave e quatro semanas para eventos hemorrágicos.^38^^,^^39^ Os resultados do estudo de García-Cabrera et al.^2^ estão de acordo com essas recomendações, embora o risco de complicações pós-operatórias fosse baixo após um pequeno evento isquêmico e, portanto, eventos menores não devem ser um impedimento para a plástica da válvula cirúrgica quando necessário.^2^

Em nosso estudo, a cirurgia foi associada à redução da mortalidade, tanto intra-hospitalar quanto em um ano. Porém, este é um estudo retrospectivo e não houve pareamento entre os pacientes que foram ou não submetidos à cirurgia, portanto não podemos concluir que a cirurgia diminui a mortalidade e podemos argumentar que esses pacientes, selecionados por uma equipe multidisciplinar, tiveram um prognóstico melhor e perfil de risco mais favorável em relação aos não submetidos à cirurgia. Deve-se enfatizar que alguns pacientes com indicação de cirurgia provavelmente foram considerados muito frágeis ou muito instáveis para o procedimento e, portanto, os resultados do presente estudo devem ser interpretados como sugerindo que a melhora do prognóstico provavelmente se deve à seleção criteriosa dos pacientes, e não à presença de indicação de cirurgia, ou à sua realização *per se*.

Além disso, em nossa coorte, a mediana do tempo até a cirurgia desde o diagnóstico de complicações neurológicas foi de 36 dias, o que está de acordo com a maioria das recomendações que apontam que deve ser adequado esperar entre duas a quatro semanas, principalmente nos casos de isquemia extensa ou derrames hemorrágicos.^1^

Ao contrário da maioria da literatura publicada, em nosso estudo as complicações neurológicas não foram associadas a um aumento significativo na mortalidade, embora a mortalidade hospitalar tenha sido numericamente maior em pacientes com complicações neurológicas (39,3 *vs.* 28,1%, p = 0,247).^13^^,^^19^ Nossa hipótese é que a associação com a mortalidade depende do tipo e gravidade das complicações neurológicas, embora a graduação padronizada da gravidade das complicações cerebrovasculares (clínicas ou radiológicas) seja fornecida em muito poucos relatos.^20^ Por exemplo, no estudo de García-Cabrera et al.,^2^ apenas eventos isquêmicos moderados a graves, particularmente hemorragias cerebrais, foram associados a um pior resultado, com complicações hemorrágicas claramente relacionadas à infecção por *S. aureus* e terapia anticoagulante, que foi usada principalmente em pacientes com próteses mecânicas.^2^

Em nossa coorte, a mortalidade hospitalar foi de 30,3%, semelhante aos dados publicados que variam de 15 a 30%.^1^ O prognóstico na EI é influenciado pelas características do paciente, a presença ou ausência de complicações cardíacas e não cardíacas, o organismo infectante e os achados ecocardiográficos, com pacientes em maior risco de insuficiência cardíaca, complicações perianulares e/ou infecção por *S. aureus.*^1^ Até onde sabemos, nenhum estudo publicado relatou os preditores de mortalidade em pacientes com EI e embolização do SNC. Em nosso estudo, o único preditor de mortalidade hospitalar e de um ano foi a infecção pelo HIV, que costuma estar associada ao envolvimento do SNC, embora não tenha sido associada a pior prognóstico nessa população. De fato, um estudo de 77 pacientes sul-africanos com endocardite, 17 dos quais infectados pelo HIV, encontrou uma taxa semelhante de complicações em pacientes com e sem infecção pelo HIV.^40^

### Limitações

Devido à natureza retrospectiva deste estudo, existem algumas limitações. Em primeiro lugar, como mencionado anteriormente, os exames de imagem da cabeça não eram realizados rotineiramente em todos os pacientes, o que pode resultar em uma subestimação da real incidência de complicações embólicas, visto que frequentemente são clinicamente silenciosos. Em segundo lugar, trata-se de um estudo observacional, com amostra relativamente pequena, e alguns resultados devem ser interpretados com cautela, a saber, a redução da mortalidade em pacientes com complicações neurológicas submetidos à cirurgia valvar, uma vez que provavelmente pacientes com complicações mais graves ou mais frágeis tinham menor probabilidade de receberem proposta de cirurgia ou tiveram a mesma negada, resultando em viés de seleção.

Por outro lado, este estudo avaliou uma coorte de uma instituição com um único centro de referência cirúrgica, sugerindo que as decisões quanto à realização e ao momento da cirurgia após o evento foram aproximadamente as mesmas.

## Conclusões

Nessa população, a embolização para o SNC era comum, mais frequentemente apresentada como AVC isquêmico, e estava associada a maior tempo de internação, embora não houvesse diferenças significativas na mortalidade. Este estudo está de acordo com dados recentes que mostram que a cirurgia deve ser a abordagem preferida em pacientes com embolização do SNC, após criteriosa seleção multidisciplinar. Também mostra que os pacientes com choque séptico e infecção pelo HIV têm um prognóstico particularmente ruim, destacando o papel da equipe de endocardite com abordagem multidisciplinar.
