# Supplementary material for: Complicações Neurológicas em Pacientes com Endocardite Infecciosa: Perspectivas de um Centro Terciário
Source: Arq Bras Cardiol. 2021 Apr 8;116(4):682–91. [Article in Portuguese] doi: 10.36660/abc.20190586 (PMC8121414; doi:10.36660/abc.20190586)
Supplement: Translation

# Translation: Neurological Complications in Patients with Infective Endocarditis: Insights from a Tertiary Centre

**Authors:** Sofia Alegria, Ana Marques, Inês Cruz, Ana Luísa Broa, Ana Rita F. Pereira, Isabel João, Otília Simões, Hélder Pereira

**Affiliations:** 1 Almada Portugal Hospital Garcia de Orta EPE – Cardiologia, Almada – Portugal.

**Keywords:** Endocarditis, Infectious/surgery, Endocarditis, Infectious/complications, Central Nervous System/complications, Stroke, Embolization, Prognosis, Mortality

## Abstract

**Background:**

Neurological complications are common in patients with infective endocarditis (IE). Recent data suggest that neurologic events are a major determinant of prognosis, and that surgery is critical in improving the outcome.

**Objective:**

To characterize patients with IE and neurological complications and to determine predictors of embolization to the central nervous system (CNS) and mortality.

**Methods:**

Retrospective analysis of patients admitted to a tertiary center with the diagnosis of IE from 2006 to 2016. Statistical significance was defined by a p-value < 0.05.

**Results:**

We identified 148 episodes of IE, 20% of which had evidence of CNS embolization. In patients with CNS embolization, 76% presented with ischemic stroke. During follow-up, 35% were submitted to surgery and both in-hospital and one-year mortality were 39%. These patients had longer hospitalizations, but there were no significant differences regarding mortality in patients with and without CNS embolization. The independent predictors of neurological complications were diabetes (p=0.005) and the absence of fever at presentation (p=0.049). Surgery was associated with lower mortality (0 vs. 58%; p=0.003), while patients with septic shock had a poorer prognosis (75 vs. 25%; p=0.014). In multivariate Cox regression, human immunodeficiency virus (HIV) infection was the only independent predictor of in-hospital and 1-year mortality (p=0.011 in both).

**Conclusions:**

In this population, embolization to the CNS was common, more often presented as ischemic stroke, and was associated with longer hospitalization, although without significant differences in mortality. In patients with CNS embolization, those submitted to surgery had a good clinical evolution, while patients with septic shock and HIV infection had a worse outcome. These results should be interpreted with caution, taking into consideration that patients with more severe complications or more fragile were probably less often considered for surgery, resulting in selection bias.

## Introduction

Neurological complications are common in infective endocarditis (IE), occurring in 15–30% of patients.^1^^–^^3^ Clinical presentation is variable and may include multiple symptoms or signs, though there is a predominance of focal signs and ischemic strokes are more often diagnosed. Transient ischemic attack, intracerebral or subarachnoidal hemorrhage, brain abscess, meningitis, and toxic encephalopathy are also observed, and firm evidence supports that additional clinically silent cerebral embolisms occur in 35–60% of IE patients.^4^^–^^6^ Sepsis-related encephalopathy, defined by acute confusion or delirium, with varying levels of consciousness, may also contribute to neurological manifestations of IE.^7^

According to this, one should always consider IE in the differential diagnosis of patients with acute cerebral event and signs of systemic infection or history of undetermined febrile syndrome, keeping in mind that early diagnosis and the implementation of adequate antibiotic treatment can reduce the risk of recurrent embolization.^1^

Risk factors for central nervous system (CNS) embolization are well-known and include vegetation size and mobility,^2^^,^^8^^–^^10^ Staphylococcus aureus infection,^11^ and mitral valve involvement.^10^ Nevertheless, the risk of CNS embolic events in IE decreases dramatically after the initiation of effective antimicrobial therapy, to less than 1.71/1,000 patient-days in the second week.^12^

Neurological manifestations occur before or at the time of IE diagnosis in a majority of cases, but new or recurrent events can also take place later in the course of IE. Neurological complications are associated with excess mortality, as well as sequelae, particularly in the case of stroke^2^^,^^13^ and affect both medical therapy^14^ and the optimal timing for surgery.^15^ Rapid diagnosis and initiation of appropriate antibiotics are of major importance to prevent a first or recurrent neurological complication.^12^ Early surgery in high-risk patients is the second mainstay of embolism prevention, while antithrombotic drugs have no function.^1^ Recent data suggest that neurological events are a major determinant of prognosis, and that surgery has a central role in improving the outcome.

However, the occurrence of neurological complications raises questions regarding the timing of surgery, as the safety of cardiopulmonary bypass in patients with these events remains controversial. The decision should be individualized after multidisciplinary assessment, involving cardiologists, cardiac surgeons, neurologists, and infectious disease specialists. If possible, surgery should be delayed in patients with large ischemic events or hemorrhagic events. It has been suggested that surgery should be considered within the first 72 hours in patients with ischemic events and severe heart failure, otherwise after four weeks. Early surgery appears safe in patients with transient ischemic attacksor silent events.

Therefore, the aim of this study was to characterize patients with IE and neurological complications and to determine predictors of embolization to the CNS and of associated mortality.

## Methods

Retrospective, observational study based on the analysis of medical records of patients admitted to a 500-bed tertiary care center without on-site cardiac surgery and diagnosed with IE from 2006 to 2016. A comparison between patients with and without neurological complications was performed. This study was approved by the Ethics Committee of the institution.

### Study Design and Patients

Several variables were analyzed for the present study, including the date of IE diagnosis; patients’ age and gender; risk factors; type of endocarditis (native valve, prosthetic valve or device-associated); valves affected; infecting microorganism; date, type, and recurrence of neurological complications; performance of surgery; and outcomes. Prosthetic valve endocarditis was considered early if it occurred within 1 year after valve implantation and late if it occurred thereafter.

IE episodes were evaluated retrospectively according to the modified Duke criteria. Only patients with criteria for definite or possible IE and no other explanation for the clinical picture were included. Relapses were considered as a single episode, while distinct episodes occurring in a single patient were included. Transthoracic echocardiography was performed in all patients, while transoesophageal echocardiography was performed in the majority of patients. Microbiological information was obtained from blood cultures and intraoperative heart tissue specimens, as well as from serological studies when blood cultures were negative.

### Definitions

Neurological complications were classified into the following categories: ischemic complications, cerebral hemorrhage, mycotic aneurism, meningitis, and brain abscess. The diagnosis of ischemic and hemorrhagic complications was based on clinical and radiological data, derived from head computed tomography (CT) or magnetic resonance imaging (MRI), performed in accordance with clinical practice. The diagnosis of mycotic aneurysm was also supported by head CT angiography.

### Indication for Surgery

The indication for cardiac surgery was determined by the attending physicians according to the guidelines of the European Society of Cardiology.^1^ All patients with indication for surgery were discussed by the cardiac team (including cardiologists, cardiac surgeons and, when deemed necessary, other specialties, such as neurologists and infectious disease specialists), and the decision about the performance and timing of surgery was made. When indicated, surgery was performed in the referral cardiac surgery center defined by the national health care system (Department of Cardiac Surgery,Santa Maria University Hospital, CHULN, Lisbon, Portugal).

### Statistical Analysis

Continuous variables are described as mean ± standard deviation or median and interquartile range (IQR), according to the assessment of normality with the Kolmogorov-Smirnov test. Categorical variables are reported as percentages and absolute numbers. A comparison between variables in different patient groups was performed with the Pearson X^2^ test for categorical variables or the independent samples t-test or Mann-Whitney U test for continuous variables. Analysis of baseline characteristics, type of endocarditis, etiology, complications, and management was performed per episode, while analysis of mortality was performed per patient.

Variables associated or with a trend to association with CNS embolization (p < 0.10) were tested with univariate and multivariate logistic regression, in order to identify independent predictors of embolization in the overall population. In the sample of patients with CNS embolization, variables associated or with a tendency to be associated with in-hospital and one-year mortality (p < 0.10) were tested with univariate and multivariate forward stepwise Cox regression, to identify independent predictors of prognosis. Kaplan Meier survival curves were used to identify predictors of outcome, which were compared with the log-rank test.

All tests were two-sided and statistical significance was defined as p < 0.05. Statistical analysis was performed using IBM SPSS Statistics, version 24.0 (IBM Corporation, Armonk, NY, USA).

## Results

We identified 148 IE episodes (occurring in 142 patients; four patients had two episodes and one patient had three episodes; relapses were considered as a single episode). The characterization of the total episodes is detailed in Table 1. The median follow-up was 161 days (IQR 34-1,358).

**Table 1:** Characterization of all episodes of admissions due to endocarditis (n=148)

Characteristic	Overall episodes (n=148)	With CNS embolization (n=29)	Without CNS embolization	p*
Age (years) – median (IQR)	64 (51-75)	65 (53-69)	63 (50-75)	0.631
Age ≤ 75 years – n (%)	117 (79.1)	27 (93.1)	90 (75.6)	0.038
Male – n (%)	111 (75.0)	22 (75.9)	89 (74.8)	0.905
**Previous history – n (%)**				
Known valvular heart disease	72 (49.0)	15 (51.7)	57 (48.3)	0.741
Arterial hypertension	76 (51.4)	16 (55.2)	60 (50.4)	0.646
Diabetes mellitus	28 (19.2)	11 (37.9)	17 (14.5)	0.004
Coronary artery disease	21 (14.2)	6 (20.7)	15 (12.6)	0.263
Heart failure	40 (27.0)	4 (13.8)	36 (30.3)	0.074
Chronic kidney disease	22 (14.9)	4 (13.8)	18 (15.1)	0.856
Intravenous drug users	19 (12.9)	3 (10.3)	16 (13.6)	0.644
HIV infection	20 (13.6)	2 (6.9)	18 (15.3)	0.240
Invasive procedure in the preceding 3 months	54 (45.0)	10 (43.5)	44 (45.4)	0.870
**Type of endocarditis - n (%)**				
Health care-associated endocarditis	34 (23.3)	9 (31.0)	25 (21.4)	0.270
Prosthetic valve endocarditis	37 (25.0)	9 (31.0)	28 (23.5)	0.403
Implanted cardiac device endocarditis	5 (3.4)	1 (3.4)	4 (3.4)	0.981
**Affected valve – n (%)**				
Aortic	84 (56.8)	19 (65.5)	65 (54.6)	0.288
Mitral	58 (39.2)	13 (44.8)	45 (37.8)	0.488
Tricuspid	20 (13.5)	0 (0.0)	20 (16.8)	0.018
**Symptoms / signs at presentation – n (%)**				
Fever	102 (70.3)	16 (55.2)	86 (74.1)	0.045
Heart murmur	81 (56.3)	14 (50.0)	67 (57.8)	0.458
**Microorganism – n (%)**				
Staphylococcus sp	49 (33.1)	8 (27.6)	41 (34.5)	0.481
Staphylococcus aureus	36 (24.3)	6 (20.7)	30 (25.2)	0.611
*Streptococcus* sp	43 (29.1)	9 (31.0)	34 (28.6)	0.793
*Streptococcus* bovis	14 (9.5)	3 (10.3)	11 (9.2)	0.856
*Streptococcus* viridans group	18 (12.2)	2 (6.9)	16 (13.4)	0.333
*Enterococcus* sp	18 (12.2)	3 (10.3)	15 (12.6)	0.738
Gram negative bacteria	6 (4.1)	1 (3.4)	5 (4.2)	0.854
Fungi	3 (2.0)	1 (3.4)	2 (1.7)	0.545
BCNIE	30 (20.3)	6 (20.7)	24 (20.2)	0.950
**Complications – n (%)**				
Perivalvular abscess	20 (14.8)	4 (14.8)	16 (14.8)	1.000
Pseudoaneurism	7 (5.2)	2 (7.4)	5 (4.6)	0.560
Fistula	6 (4.4)	1 (3.7)	5 (4.6)	0.835
Acute heart failure	71 (48.0)	15 (51.7)	56 (47.1)	0.652
Septic shock	31 (20.9)	8 (27.6)	23 (19.3)	0.327
**Treatment**				
Surgery – n (%)	48 (32.4)	10 (34.5)	38 (31.9)	0.793
Duration of hospitalization (days) – median (IQR)	40 (26-54)	51 (36-59)	38 (25-52)	0.011

* comparison between patients with and without CNS embolization. CNS: central nervous system; IQR: interquartile range; HIV: human immunodeficiency virus; BCNIE: blood culture negative infective endocarditis.

About one-third of them (34.5%; n = 51) presented evidence of systemic embolization, and the most frequent site was the CNS (19.6%; n = 29). Other embolization sites included peripheral circulation (4.1%, n=6), lungs (2.7%, n=4), coronary arteries (1.4%, n=2), and spleen (1.4%, n=2). Nevertheless, only 34.5% (n = 51) performed head CT or MRI, so the true incidence of CNS embolization could be underestimated. Considering only patients with left-sided IE, the incidence of CNS embolization was 24.2% (n=29). The characterization of patients with a diagnosis of CNS embolization is also detailed in Table 1. These patients were predominantly male, with a median age of 65 years; 48.3% had previously known valvular disease, 10.3 % were intravenous drug users, and 6.9% had human immunodeficiency virus (HIV) infection. Native valve endocarditis was more common (69.0%, n = 20), while prosthetic valve endocarditis occurred in 31.0%, with 33.3% of prosthesis (n = 3) implanted in the preceding 12 months.

Among patients with HIV infection, 47.4% (n = 9) were treated with antiretroviral therapy, the median CD4 count was 202.5 cells/µl (interquartile range 10 – 402.5 cells/µl), 62.5% (n = 10) had undetectable viral load (median viral load of 0 copies/ml; interquartile range of 0 – 3,127 copies/ml), and 46.7% (n = 7) had criteria for acquired immunodeficiency syndrome (AIDS).

Patients with CNS embolization presented with ischemic stroke in 75.9% (n = 22) of cases (with hemorrhagic transformation in 27.3% of them; n = 6), hemorrhagic stroke in 17.2% (n = 5), mycotic aneurism in 17.2% (n = 5), and meningitis in 3.4% (n = 1). Upon admission, neurological symptoms were present in 41.4% (n = 12), and there were stroke recurrences (including both ischemic and hemorrhagic) in 34.5% (n=10) (Figure 1).

**Figure 1:** Neurological complications in patients with endocarditis (n=29).

### Predictors of CNS Embolization

Patients with CNS embolization, compared to those without it, were more likely to be younger than 75 years, to have diabetes, and to present without fever (Table 1). In addition, no patients with CNS embolization had involvement of right-sided valves, and they had longer hospitalization periods (median 51 vs. 38 days). There were no significant differences regarding the etiologic agent, the involvement of aortic or mitral valve, the proportion of patients submitted to surgery, orthe outcome.

In multivariate logistic regression, the independent predictors of CNS embolization were diabetes and the absence of fever (hazard ratio – HR 3.78, and 2.41, respectively) (Table 2).

**Table 2:** Predictors of central nervous system embolization

Characteristic	HR	95% CI	p
Diabetes mellitus	3.8	1.5-9.6	0.005
Absence of fever at presentation	2.4	1.0-5.8	0.049

HR: hazard ratio; CI: confidence interval.

### Outcomes in Patients with Neurological Complications

During follow-up (median 493 days, IQR 36-1,863), 34.5% of patients with CNS embolization (n = 10)were submitted to surgery. The median time from admission to surgery was 41 days (IQR 33-55) and fromthe diagnosis of the neurological complication to surgery was 36 days (IQR 28-43). Both in-hospital mortality and 1-year mortality were 39.3% (n=11) and all-cause mortality during follow-up was 46.4% (n=13) (Table 3).

**Table 3:** Mortality of patients with endocarditis (n=142)

Characteristic – n (%)	Overall population (n=142)	Patients with CNS embolization (n=29)	Patients without CNS embolization (n=113)	p*
In-hospital mortality	43 (30.3)	11 (39.3)	32 (28.1)	0.247
One-year mortality	55 (38.7)	11 (39.3)	44 (38.6)	0.947
Overall mortality	64 (45.1)	13 (46.4)	51 (44.7)	0.872

* comparison between patients with and without CNS embolization. CNS: central nervous system.

Surgery was associated with reduced mortality, both in-hospital and at 1 year (mortality at 1 yearin patients submitted to surgery: 0 vs. 57.9%; p=0.002). In addition to surgery, the other variables associated with in-hospital mortality were the occurrence of septic shock and invasive procedures in the preceding three months (Table 4). In multivariate Cox regression analysis, HIV infection was the only independent predictor of both in-hospital and one-year mortality (HR 10.5 and 10.6, respectively) (Tables 5 and 6, Figure 2).

**Table 4:** Associations with in-hospital mortality in patients with central nervous system embolization

Characteristic	OR	95% CI	p
Diabetes *mellitus*	3.9	0.8-20.0	0.094
HIV infection	N/A	N/A	0.068
Invasive procedure in the preceding 3 months	4.5	0.7-27.7	0.096
Septic shock	9.0	1.4-59.8	0.014
Surgery	N/A	N/A	0.003

OR: odds ratio; CI: confidence interval; HIV: Human immunodeficiency virus; N/A: not applicable.

**Table 5:** Independent predictors of in-hospital mortality in patients with central nervous system embolization

Characteristic	HR	95% CI	p
HIV infection	10.5	1.7-64.2	0.011

HR: hazard ratio, CI: confidence interval; HIV: human immunodeficiency virus.

**Table 6:** Independent predictors of one-year mortality in patients with central nervous system embolization

Characteristic	HR	95% CI	p
HIV infection	10.6	1.7-64.8	0.011

HR: hazard ratio; CI: confidence interval; HIV: human immunodeficiency virus.

**Figure 2:** Kaplan-Meier survival curve of patients with endocarditis and neurological complications according to the status of human immunodeficiency virus infection. HIV: Human immunodeficiency virus.

## Discussion

This retrospective observational study describes the incidence of neurological complications in a cohort of patients with IE from a single center during a 10-year period.

Neurological complications are a common and often predominant feature of IE^3^^,^^13^^,^^16^^–^^18^ and the advent of CT and MRI enables a more reliable clinical assessment of these events. However, there are few available data about the risk of recurrent stroke, the best approach with regard to antithrombotic therapy, or the consequences of early surgery.^2^

The overall frequency of neurological complications in the present study cohort was around 20%, keeping up with the results from other large cohorts.^19^^,^^20^ In the present study, it was also found that older patients had lower rates of these events, as previously reported,^19^^,^^21^ although the cause of this reduction is not fully understood. Use of antiplatelet therapy^22^^,^^23^ (often prescribed in aged patients), a hypothetical decline in hemostatic function, and smaller size of vegetations in this population are some of the reasons proposed,^17^ but it is also possible that these events are simply underdiagnosed in this population due to mild clinical signs and symptoms.

Head imaging exams were not routinely performed in all patients, and the true incidence of ischemic complications is probably underestimated in the present cohort. Studies using MRI^6^^,^^24^ have shown that acute brain embolizations are significantly more prevalent than it has been previously reported in studies based on clinical findings and CT scanning (30% of undetected events). With this taken into account, it is possible that some less symptomatic aged patients in the present study have been wrongly classified as having no neurological complications. However, other reports^20^^,^^25^ have shown that small ischemic complications have no impact on the outcome of patients with IE, and, therefore, the essential conclusions would not be changed.

In the present study, the predictors of CNS embolization were a history of diabetes mellitus and the absence of fever at presentation. Several studies have shown mitral valve involvement and vegetation size to be important predictors of stroke,^10^^,^^17^^,^^19^^,^^26^^–^^29^ whereas others have not confirmed this observation.^29^^–^^32^ In the present cohort, mitral valve involvement was not associated with neurological complications. Vegetation size was not assessed, since measurements were not available for all patients and also due to a lack of standardization of the existing measurements. Some authors have emphasized the importance of vegetation size only when other factors are present, such as mitral valve location, and Staphylococcus aureus as the etiologic agent of IE.^30^^–^^33^ In this cohort, it is possible to hypothesize that the influence of vegetation location and size on the development of embolic events was probably outweighed by factors that lead to a delay in the diagnosis and initiation of antibiotic therapy, such as the absence of fever at presentation. To our knowledge, diabetes mellitus has not previously been identified as a risk factor for CNS embolization in patients with IE, although it is associated with a worse prognosis in IE.^1^ Nevertheless, diabetes is associated with an increased risk of cerebrovascular events and immunosuppression, so we can speculate that this condition may facilitate the growth of vegetations and increase the severity and clinical impact of embolization, when this complication occurs.

The timing of surgery in these patients is still a source of debate. Prompt surgery to prevent embolic events based on a vegetation size above 10 millimeters was proposed in early echocardiographic studies,^34^ but higher rates of relapse and prosthesis dehiscence after surgery when antimicrobial treatment has not been completed remain a concern. In this regard, two recent studies have demonstrated that early surgery effectively decreases systemic embolism without increasing the IE relapse rate or prosthetic valve-related problems compared with the conventional treatment.^35^^,^^36^

Likewise, there is concern about the risk of postoperative neurological impairment when valve surgery is performed early after an ischemic or hemorrhagic episode, and the literature contains contradictory results. Some authors have found the risk of exacerbation to be low when surgery was performed within 72 hours,^37^ whereas others have reported that the risk is higher in early surgery and gradually decreases as the delay between the neurological event and the operation increases.^38^ Considering the lack of controlled studies, recommendations are based on the results of published reports, and the generally accepted advice is to delay surgery for at least two weeks in the case of severe ischemic strokes and four weeks for hemorrhagic events.^38^^,^^39^ The results of the study by García-Cabrera et al.^2^ are in line with these recommendations, although the risk of postoperative complications was low after a small ischemic event, and therefore, minor events should not be an impediment to surgical valve repair when necessary.^2^

In our study, surgery was associated with reduced mortality, both in-hospital and at one year. However, this is a retrospective study and there was no matching between patients who were submitted to surgery or not, so we cannot conclude that surgery decreases mortality and can argue that these patients, selected by a multidisciplinary team, had a better prognosis and a more favorable risk profile compared to those not submitted to surgery. It should be emphasized that some patients with indication for surgery were probably considered too fragile or too unstable to undergo surgery, thus the results of the present study should be interpreted as suggesting that the improvement of prognosis is likely due to the careful selection of patients, and not to the presence of an indication for surgery, or to the performance of surgery per se.

Additionally, in our cohort, the median time to surgery since the diagnosis of neurological complications was 36 days, which is in line with most of the recommendations that point out that it should be appropriate to wait between two to four weeks, especially in extensive ischemic or hemorrhagic strokes.^1^

Contrary to most of the published literature, in our study, neurological complications were not associated with a significant increase in mortality, although in-hospital mortality was numerically higher in patients with neurological complications (39.3 vs. 28.1%, p=0.247).^13^^,^^19^ We hypothesize that the association with mortality depends on the type and severity of neurological complications, although a standardized grading of the severity of cerebrovascular complications (clinical or radiological) is provided in very few reports.^20^ For instance, in the study by García-Cabrera et al.^2^ only moderate-to-severe ischemic events, particularly cerebral hemorrhages, were associated with a worse outcome, with hemorrhagic complications clearly related to S. aureus infection and anticoagulant therapy, which was mainly used in patients with mechanical prosthesis.^2^

In our cohort, in-hospital mortality was 30.3%, similar to published data ranging from 15 to 30%.^1^ Prognosis in IE is influenced by patient characteristics, the presence or absence of cardiac and non-cardiac complications, the infecting organism and the echocardiographic findings, with patients with heart failure, periannular complications and/or S. aureus infection at highest risk.^1^ To our knowledge, no published study reported the predictors of mortality in patients with IE and CNS embolization. In our study, the only predictor of both in-hospital and one-year mortality was HIV infection, which is often associated with involvement of the CNS, although it has not been associated with a worse prognosis in this population. Indeed, a study of 77 South African patients with endocarditis, 17 of which were HIV-infected, found a similar rate of complications in patients with and without HIV infection.^40^

### Limitations

Due to the retrospective nature of this study, there are some limitations. First, as previously mentioned, head imaging exams were not routinely performed in all patients, which may result in an underestimation of the true incidence of embolic complications, since they are frequently clinically silent. Second, this was an observational study, with a relatively small sample, and some results should be interpreted with caution, namely the reduction in mortality in patients with neurological complications submitted to valve surgery, since more fragile patients or with more severe complications were probably less likely to be proposed or were refused surgery, resulting in selection bias.

On the other hand, this study assessed a cohort from an institution with a single surgical referral center, implying that the decisions regarding the performance and timing of surgery after the eventwere approximately the same.

## Conclusions

In this population, embolization to the CNS was common, more often presented as ischemic stroke, and was associated with longer hospitalization, although there were no significant differences in mortality. This study is in line with recent data that showed that surgery should be the favored approach in patients with CNS embolization, after careful multidisciplinary selection. It also shows that patients with septic shock and HIV infection have a particularly poor prognosis, highlighting the role of the endocarditis team with a multidisciplinary approach.
